# Deliberations on the microplastic-guided rare heavy metal toxicity in agricultural crops grown around nuclear reactors: molecular regulation and strategies for remediation

**DOI:** 10.1007/s44297-026-00066-7

**Published:** 2026-02-25

**Authors:** Aditya Banerjee

**Affiliations:** Department of Molecular, Cellular and Developmental Biology, University of Michigan, Ann Arbor, MI USA

**Keywords:** Active transport, Crops, Microplastics, Plant growth regulators, Phytoremediation, Rare heavy metals, Toxicity

## Abstract

Rapid growth in the nuclear energy sector has led to increased construction of nuclear power plants (NPPs). Although this promotes the generation of alternate sources of “clean” energy that does not harm the environment, potential concerns regarding soil and water pollution with microplastics and multiple rare heavy metals (HMs) used in NPPs usually do not grab the required attention. The group of rare HMs comprises of uranium, cadmium, mercury, cobalt, germanium, and indium, which are known ecological toxins affecting agricultural quality and consumer safety. When expunged as nuclear waste discharges, these rare HMs adsorb to the surface of microplastics and together pollute the adjacent cultivable lands and water sources used for irrigation. Microplastics increase the phyto-availability of the HMs, which mimic micronutrient elements and are actively transported into root cells via calcium, iron, zinc, copper, or other HM transporters. The toxicants are then translocated to aerial biomass and reproductive or storage organs via the symplastic or apoplastic routes. Humans or animals consuming such contaminated crops and vegetables can develop irreversible neurological and physiological disorders, including cancers. Plant growth regulators like abscisic acid, gibberellic acid, and nitric oxide have been found to synchronize the stress-adaptive signaling in crops, although the sensitive genotypes ultimately succumb to oxidative injuries. To abate such ecological and economic loss, remote sensing can be used to avoid contaminated areas or bio(phyto)remediation can be performed to depollute contaminated landscapes and water bodies. Genetically engineered, tolerant crops can also be cultivated directly, with lower yield loss.

## Introduction

Heavy metals (HMs) used in nuclear reactors and power plants exert severe radiotoxicity as well as chemotoxicity when discharged into adjacent agricultural lands or water bodies as wastes [1]. Especially after the Fukushima and Chernobyl disasters, adjacent crops have been found to be severely afflicted and polluted with nuclear waste discharges, including radionuclides [2]. Depending on the agricultural conditions, any crop can be cultivated near nuclear reactors; the majority of these are corn and soybean crops across the Midwestern United States [3]. These HMs include uranium (U), cadmium (Cd), indium (In), germanium (Ge), cobalt (Co), mercury (Hg) etc., which can easily enter the food chain through soil-crop transfers and cause irreversible physiological syndromes and cancers in human and animal consumers [4]. This review refers to these HMs as rare because of their low availability, and especially of the radionuclide forms in the environment. The rare radioactive HM, U^238^, is used in nuclear reactors as fuel for producing nuclear electricity and radioactive weapons [5]. Cd is used to make the control rods, which are used to regulate the nuclear chain reaction rate after insertion into the core of the reactor [6]. The HM In is used to monitor thermal neutron flux within reactors [7]. Ge^74^ is widely used to construct semiconductor detectors, radiation monitors, control rods and isotope labeling reactions [8]. The radionuclide Co^60^ is produced for medical applications like irradiation and radiation processing [9]. Unstable isotopes of Hg have been routinely used for research investigations in nuclear reactors worldwide, European Organization for Nuclear Research being one of them [10]. Severe pollution of groundwater, used for irrigation, with rare HMs and radionuclides of cesium (^137^Cs), radium (^228^Ra, ^226^Ra) and potassium (^40^K) has been reported in the surroundings of the Rooppur nuclear power plant (NPP) in Bangladesh [11]. Co-contamination of nuclear wastes containing HMs has also been confirmed in the water sources of Northwest Nigeria [12]. Hence, agricultural pollution from rare HMs in NPP discharges is a rising ecological concern that directly challenges food security and quality of life of the consumers.

Rare HMs released from nuclear reactors are usually toxic xenobiotics that are imported into the plant roots via transporters involved in the uptake of physicochemically related beneficial metals [13]. Bioaccumulation triggers overproduction of reactive oxygen species (ROS) like superoxide radicals (O_2_^−^), hydrogen peroxide (H_2_O_2_), hydroxyl radicals (OH^−^), etc., which imposes cellular oxidative stress, resulting in genotoxicity, enzymatic inactivation, necrosis, and reduced yield in glycophytic crops [14–17]. Plant growth regulators like phytohormones, gasotransmitters, and defense biomolecules such as antioxidants, are known to play crucial regulatory roles in response to such HM stress [18, 19]. Some wild plants sequester the HMs through tissue or organellar compartmentalization, for which these can tolerate them at high levels [20]. These plants sometimes release organic exudates in the rhizosphere to chelate and immobilize the toxicants, and hence can be used for phytoremediation and depollution of contaminated agricultural fields [21, 22].

Plastic particles with less than 5-mm dimensions are referred to as microplastics (MPs), and these emerging pollutants have become ubiquitous in the ecosystem due to extensive industrialization and unplanned disposal of plastic wastes or products [23, 24]. Recent studies are showing that along with rare HMs, NPPs also release MPs across the adjacent areas and severely affect community structure and dynamics [25]. This also affects agriculture, since deleterious effects of MPs have been reported in many important crop species. It has been observed that MP toxicity inhibited auxin signaling in wheat seedlings and impeded the formation of adventitious roots that govern uptake of minerals and nutrients [25]. Furthermore, the presence of polystyrene MPs is also known to hinder *cis*-zeatin riboside (cytokinin) signaling in wheat roots and jasmonic acid–dependent lignin biosynthesis and cell wall strengthening in rice roots [26–28]. Hence, remote identification of contaminated sites followed by sustainable removal of MPs via bioremediation is necessary to depollute agricultural fields. However, the chemical nature of MPs spewed by reactors still needs to be investigated.

Alarmingly, rare HMs can piggyback on MPs through surface adsorption when co-existing in soil or water bodies. According to the 2022 U.S. Department of Energy report, almost 2,000 metric tons of spent fuel is generated every year from NPPs. Additionally, under-construction NPP sites have been found to contain 0.33 items g^−1^ of MPs, which is tenfold more than distant areas [29]. Since NPPs release both rare HMs and MPs in significant amounts, such co-action of both classes of emerging pollutants is a regular occurrence in adjacent agricultural fields or water sources that are used for irrigation. Hence, the aim of this text is to delineate the molecular co-impact of MPs and rare HMs, spewed as nuclear wastes, on agricultural sustainability and to identify eco-friendly blueprints for safe cultivation of crops in these co-contaminated areas.

## The physicochemical interaction between MPs and HMs in agricultural sites around nuclear reactors

MPs are indelible pollutants that have dramatically increased in water bodies due to the ubiquitous anthropogenic use of plasticwares in all heavy-, medium-, and small-scale industries [30]. MPs are heterogenous in nature and are usually < 5 mm in dimension [31]. Multiple reports have established the exorbitantly high MP concentrations in agricultural soils throughout the world [32]. Several physicochemical parameters like acidity, concentration of ions, etc., account for the chemical speciation of MPs and their interaction with HMs in soil [33]. HMs can adhere to MPs through various physicochemical processes, primarily electrostatic forces between charged metal ions and the MP surface, and also via surface complexation, π-π interactions, and hydrogen bonding, enhanced by the development of oxygen-containing functional groups as MPs weather. A recent investigation established a tenfold higher abundance of MPs in the soil-sediments of 20 sites near an NPP in Northeast China compared with those in the distant sites [29]. This directly verifies the extensive release of MPs from newly constructed NPPs, which together with other HMs, possess the capacity to destroy the rhizospheric and plant communities. Due to weak interactive effects characterized by dipole-cation, hydrogen bonding, and weak dipole interactions, polyamide (PN6) MPs exhibited very strong adsorption with U^232^, which often escapes as nuclear waste and gets deposited in adjacent agricultural fields [34]. The adsorption partition co-efficient was as high as 2,670 L kg^−1^. Alarmingly, the highest adsorption efficiency of the radioactive HMs U^232^ and americium-241 (Am^241^) with MPs like polyurethane and polylactic acid was at neutral or near alkaline pH, which closely resembles that of soil and water bodies [34]. Bioavailability of Hg^2+^, another HM released as a NPP waste, significantly increased in red soils polluted with polyvinyl chloride MPs due to reduction of Hg within the soil [35]. Thus, MP pollution emanating from NPPs behave as a “Trojan horse” for rare HM co-toxicity in surrounding agricultural fields.

## Uptake of MPs and active transport of rare HMs in crops

### Uptake of MPs

Irrigation with contaminated water is the primary source of MP bioavailability in plants [36], and as discussed earlier, MP pollution increases around under-construction NPPs due to release of shredded plastic wastes and exhausts. Deposition of MPs in the rhizosphere enables its effective interaction with rare HMs, also deposited as a result of unprocessed nuclear waste disposals. Usually, MPs with dimensions < 100 nm are readily taken up by plants, whereas larger MPs adhere to the outer surface of roots and release the piggybacked HMs there [37]. MPs made up of polystyrene and polymethyl methacrylate are absorbed by root hairs in plants, and these enter “the crack-entry route at the emergence of the adjacent roots” to be localized in the stellar regions of important crops like lettuce and wheat [36, 38, 39]. Polystyrene MPs are often translocated to the aerial biomass of rice, a staple food crop. Since uptake of MPs occurs via both apoplastic and symplastic routes, it jeopardizes overall plant-water-soil transit, resulting in stunted growth and reduced nutrient absorption [36]. The overall water flow is obstructed in lettuce seedlings since uptake of polystyrene MPs occurs along the transpiration pathway after they are trapped by the hydrated mucilage of the root cap, associate hydrophobically with the surface of roots, and are consumed via endocytosis [40]. This observation is more precise for dicotyledonous crops since quantitative tracing of MPs using lanthanide chelates has validated higher bioaccumulation of MPs in dicots like lettuce compared with monocots like wheat [41].

### Active transport of U

**Fig. 1:**
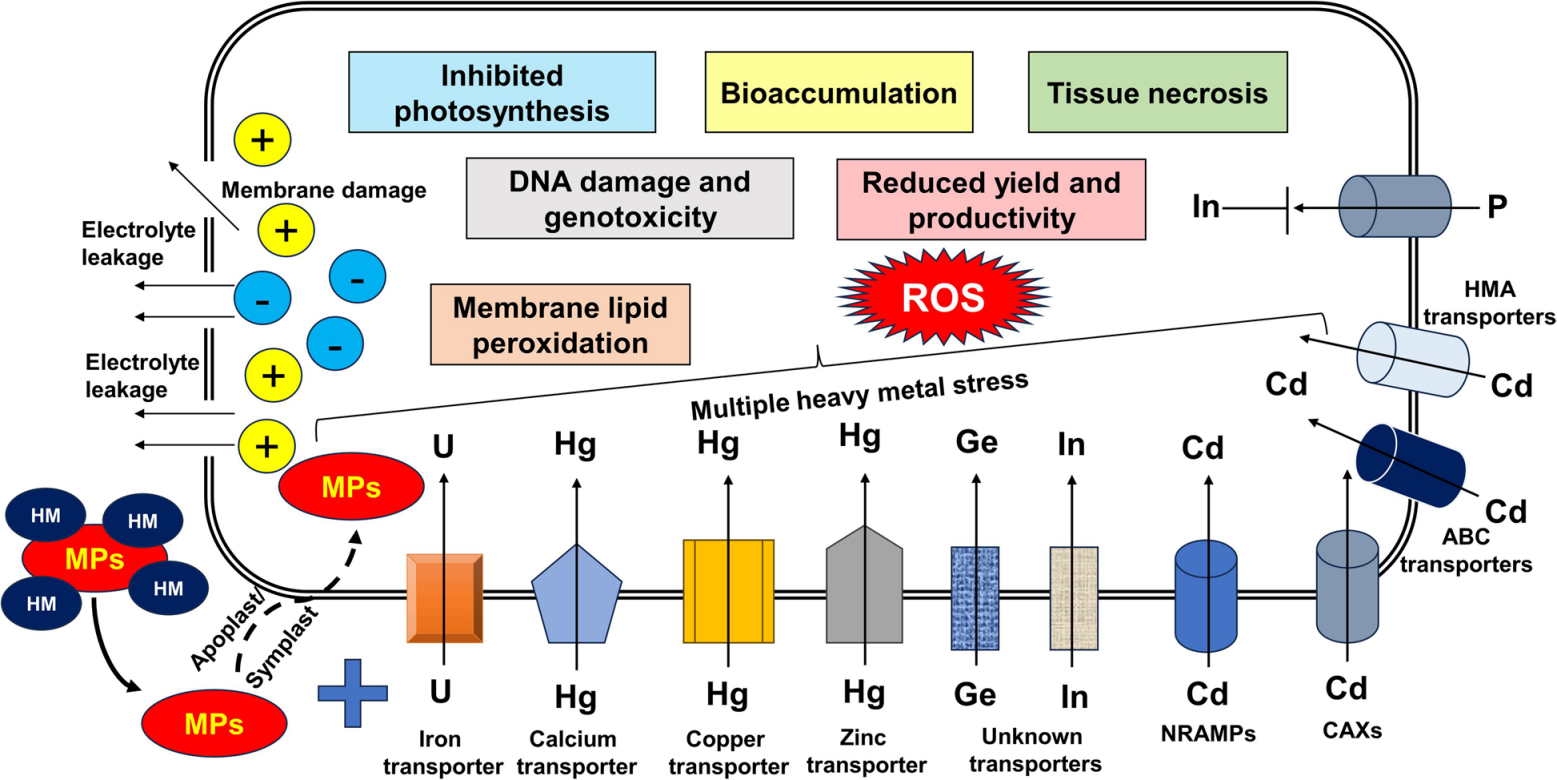
Transport and toxicity of MPs and rare HMs in plants. In co-contaminated areas, HMs are adsorbed to the surface of MPs, resulting in increased bioavailability to plant roots. The MP-HM complex dissociates before entry into plant roots. MPs enter the root hairs via the apoplastic or symplastic route, whereas HMs are actively transported via transporter proteins that usually mediate the uptake of micronutrients. Uranium is imported into root cells through iron transporters, whereas mercury is transported via calcium, copper, and zinc transporters. Cadmium is mobilized through NRAMPs, CAXs, and ABC and HMA transporters. Specific transporters involved in indium and germanium uptake have not yet been identified. Altogether, copresence of these xenobiotics triggers the production of ROS, which promote lipid peroxidation of cell membranes, resulting in leakage of crucial ions and electrolytes. Alongside that, photosynthetic efficiency is affected due to MP and HM bioaccumulation and chloroplast membrane damage. Excessive generation of ROS also triggers DNA damages, including double-strand breakages, resulting in genotoxicity and cell death. Some HMs like indium also inhibit the uptake of important nutrients like phosphorus by suppressing the expression or activity of phosphate transporters, resulting in nutrient deficiency along with severe oxidative stress. ABC: ATP-binding cassette; CAXs: cation/H + exchangers; DNA: deoxyribonucleic acid; HMA: heavy metal ATPase; HMs: heavy metals; MPs: microplastics; NRAMPs: natural resistance-associated macrophage proteins; ROS: reactive oxygen species

Uptake of rare HMs can occur via active transport in plant roots. Translocation of U has been found to be mediated by iron-regulated transporter 1 (IRT1), ferric-chelate reductase 2, iron deficiency-induced transcription factor 1, and via interactions with other metal transporters [42, 43] (Fig. 1). U can be transported via symplastic stellar route or through the xylem pathway, as UO_2_-citrate or UO_2_-lactate chelates driven by the transpiration pull [44–46]. Active transport allows the mobilization of U chelates across the Casparian strip, after which the symplastic transport is facilitated within the xylem [47, 48]. Alarming levels of U bioaccumulation have been observed in the root and aerial biomass of important crops like mustard, sunflower, garden peas, bean, maize, and sweet potato (Table 1).

**Table 1:** Enzymes used by different microbial species to degrade microplastics

Species	Enzyme	Microplastic	Reference
*Actinomycetes*	Laccase	Polyethylene	[49–51]
*Aspergillus flavus*			
*Pleurotus ostreatus*			
*Pseudomonas aeruginosa*	Alkane hydroxylase	Polyethylene	[52]
*Lentinus tigrinus*	Esterase	Polystyrene	[53]
*Ideonella sakaiensis*	Polyethylene terephthalate hydrolase, mono (2-hydroxyethyl) terephthalic acid hydrolase	Polyethylene terephthalate	[54]
*Streptomyces scabies*	Suberinase	Polyethylene terephthalate	[55]

### Active transport of Hg

Due to their lipophilic nature, monomeric and organic Hg cannot diffuse through the cell membrane in rice roots and hence are actively transported via calcium, copper, or zinc channels after being reduced to Hg^0^, after which it is mobilized through the symplastic route [56–58] (Fig. 1). It has been reported that Hg stress-activated calcium (Ca^2+^) channels in rice root membranes [59]. Methylation of inorganic Hg to its methylated form (MeHg) stimulated absorption via roots and translocation to the aerial biomass, whereas atmospheric Hg, absorbed by the leaves after deposition was not transported back to the soil nor released as volatiles, resulted in increased bioaccumulation in the shoot [60, 61]. Alarmingly, it was found that although inorganic Hg remained localized in the pericarp and aleurone layers of brown rice grains, MeHg could form a complex with cysteine and translocate to the endosperm; resultingly, MeHg could not be removed by seed polishing as can be done for inorganic Hg [62, 63]. This incurs a direct threat to consumer health because rice is the staple food crop across Southeast Asia, and rice straws are extensively used as cattle fodder due. This means Hg continues to be biomagnified in the food web [64].

### Active transport of Co

Co forms a noncationic complex with low molecular weight organic acids like malic, oxalic, citric, fumaric, and malonic acids, after which the complex is imported into root cells for long-distance translocation of the HM via the xylem, without significant cation exchange, and finally into foliar vacuoles [65–68]. Unlike other HMs, Co is transported relatively slower, through the phloem. High bioaccumulation of Co has been observed in important food crops like tomato, soybean, oilseed rape, and chickpea (Table 1). Some energy-coupling factor transporters are encoded by the *cbiQO*-like genes in plant genomes, for which they have been annotated as Co transporters [69].

### Active transport of Cd

Cd enters the cell membrane of the root epidermis either via cation exchange with H^+^ or as chelated non-ionic complexes with organic materials, after which it is transported through both the apoplast and symplast. Transporters that actively accelerate the uptake of rhizospheric Cd are zinc/iron-regulated transporters like IRT1, metal tolerant protein 1, natural resistance-associated macrophage proteins (NRAMPs), P-type ATPases like heavy metal ATPase 4 and heavy metal ATPase 9, ATP-binding cassette (ABC) transporters, and cation/H^+^ exchanger (CAX) family channel proteins like CAX2 and CAX5 [70–76] (Fig. 1). Translocation of Cd from roots to aerial biomass can occur via NRAMP1/5/6 in rice, low-affinity cation transporter-1, and the ABC transporter PDR8 [77–81]. Excess cellular Cd is chelated by reduced glutathione and phytochelatins and compartmentalized into vacuoles. This transport across the tonoplast membrane is mediated mainly by ABCs, CAXs, NRAMP3, and NRAMP4 [82–84]. Siphoning excess Cd as chelate complexes into the vacuoles is a signature of plant-HM adaptation. However, Cd bioaccumulation is a severe concern in case of edible food crops (Table 1) since their consumption results in irreversible neurological and physiological disorders in humans and animals [85].

### Active transport of In

Like other rare HMs, uptake of In is probably mediated via channels associated with iron, zinc, sodium, and magnesium transport in rice roots [86]. At toxic concentrations, In interacts with divalent micronutrient cations and impedes their uptake via competition or formation of complexes like In-ferrihydrite and In-manganese hydroxide [87]. Treatment of In-promoted phosphate deficiency in plants is done by downregulating expression of *phosphate transporter 1:1/4* and *phosphate 1*, which are associated with absorption of phosphate and xylem loading. Interestingly, no role of *IRT1* in In uptake was determined from transgenic studies using mutant lines of the gene [88].

## Phytotoxicity exerted by MPs and HMs

### Toxicity rendered by MPs alone

Bioaccumulation of MPs causes widespread genotoxicity and necrosis, especially in the delicate root tissues [89]. Polystyrene MPs have been reported to promote mitotic instability by increasing the occurrence of micronuclei in roots, resulting in mutations and aberrations in chromosomes. Such abnormal cell cycle events have been linked to regulated expression of genes like *cdc2* (encoding cyclin-dependent kinases) during MP stress [88–91]. Increased oxidative stress and cytotoxicity was observed in germinated onion seedlings exposed to a dose as low as 0.01 g L^−1^ of polystyrene MP solution for three days [92]. This exemplifies the toxic effect of MPs in crops that are continuously irrigated with contaminated water throughout their life cycle. Exposure to high concentrations of polylactic acid MPs significantly reduced the shoot, root, and biomass development in the food crop (maize), revealing its high phytotoxic effect [93]. Such decrease in growth has been attributed to reduced levels of osmolytes like proline, carotenoid, and soluble sugars, as well as an overall increase in chlorosis [94]. Degeneration of chlorophyll upon exposure to MPs is a result of excessive ROS production via cytochrome p450-mediated oxidation [95]. Studies involving carbon nanoplastics in crops like soybean, maize, and rice have shown that activity of enzymatic antioxidants increased in these species in an attempt to reverse the excessive cellular oxidative load [96]. Polystyrene MPs jeopardized crucial metabolic pathways pertaining to amino acid, fatty acid, nucleic acid, and secondary metabolite biosynthesis in rice, which directly explains the reduced energy expenditure and wilting of seedlings out of MP toxicity [95].

### Toxicity rendered by rare HMs alone

Rare HMs trigger phytotoxicity by incurring ROS production, which causes lipid peroxidation in cell membranes and genotoxicity [97] (Fig. 1). Exposure to U reduced the biomass and growth rates in broad bean, radish, cabbage, spinach, garden peas, and sweet potato, largely due to increased oxidative injuries manifested by reduced photosystem II efficiency and photosynthetic rate and the formation of malondialdehyde and reactive nitrogen species [98–105]. Based on physiological evolution and adaptation in the course of domestication, some crops might be more sensitive to particular HMs. Likewise, a comparative study revealed that maize was more sensitive to Hg stress than wheat due to higher bioaccumulation, resulting in stunted growth, decreased lignin biosynthesis, and inhibition of enzymes like phenylalanine ammonia lyase, 4-coumarate CoA ligase, and cinnamyl alcohol dehydrogenase belonging to the phenylpropanoid and monolignol pathways [106]. Cellular toxicity of MeHg and inorganic Hg—depicted by the appearance of necrotic lesions, stunting, disrupted metabolism, and compromised productivity—has been widely reported in rice [107, 108]. High concentrations of Co and Cd also accelerate cell death, genotoxicity, chlorosis, and membrane peroxidation, and they inhibit germination [109]. Cd also stalls the synthesis of zinc finger proteins, HMA9 (in rice), NO synthase activity (in pea), and photosynthesis due to exorbitant production of ROS via nicotinamide adenine dinucleotide phosphate oxidase [110, 111]. Agricultural plants severely affected by Cd toxicity are pea, wheat, alfalfa, maize, rice, barley, tomato, and spinach [112–117]. The intensity of Cd-triggered oxidative stress is so prominent in crops that it can be monitored using near-infrared second-window (NIR-II) fluorescence imaging via an activable nanoprobe or via hyperspectral imaging (HSI), and these detection methods can be used in fields to identify regions afflicted with Cd stress [118, 119].

High concentrations of inorganic and organic Ge significantly reduced biomass and apical growth in lettuce seedlings due to high bioaccumulation in roots (Table 1) [120, 121]. In toxicity also severely compromised root and shoot development along with biomass formation in rice seedlings. Additionally, anatomical perturbations like thickening of the epidermis and exodermis, lignification of sclerenchyma, protoplast depletion in roots, and development of chromoplasts in mesophyll cells due to nutritional deficiency of mainly phosphate, were visible [86, 122] (Fig. 1). In stress profoundly activated programmed cell death in wheat roots by promoting deoxyribonucleic acid (DNA) fragmentation and the number of terminal deoxynucleotidyl transferase dUTP nick end labeling–positive nuclei and by suppressing root activity [123]. As a defense response, some plants like *Arabidopsis* release organic acids like citrate to chelate in order to avoid In uptake since it inhibits phosphate transporters and triggers phosphate deficiency along with oxidative stress and anthocyanization [88].

### Cophytotoxicity of MPs and rare HMs released from NPPs

As evident from their individual phytotoxic capacities, co-exposure to both HMs and MPs further potentiates the physiological deterioration in plants [124]. Abundance of MPs affects the physicochemical properties of HMs and reduces their soil adsorption capacity, resulting in increased bioavailability to plant roots [32]. In line with this notion, presence of high-density MPs of polyethylene and polystyrene triggered Cd bioaccumulation in maize and wheat seedlings, respectively leading to cophytotoxicity and reduced productivity [125, 126]. Compromised productivity is usually due to stalled growth, as depicted by reduced diameter of the stem and root elongation in strawberry seedlings co-exposed to MPs and Cd [127]. Furthermore, HMs and MPs negatively affect germination index, plant respiration, photosynthetic efficiency, and biomass growth [128]. On the contrary, in some conditions, MPs may also enhance the soil adsorption of certain metals, which in turn reduce the uptake and toxicity of HMs in plants [129].

Phytotoxicity also involves undesirable situations like impeded mineral nutrition, resulting in seedling wilting and death. Copresence of polyvinyl chloride MPs and MeHg affect iron and sulfate uptake and nutrition in rice fields [35]. Cd-polluted soils contaminated with microfibers of polyesters decrease amino acid, sugar alcohol, carbohydrate, and nitrogen metabolism in plants grown in them [130]. Such pernicious changes in overall plant metabolism in soils copolluted with Cd and MPs might be due to altered cation exchange capacities and increase in soil acidity, which altogether incurs rhizospheric hormesis across diverse microbial communities, some of which are directly correlated with plant survival and propagation [131].

## Molecular impact of rare HM toxicity on prominent growth regulators

### Abscisic acid (ABA)

Osmotic-responsive signaling pathways regulate the “universal” stress phytohormone, ABA, and they are activated upon HM stress, as evident in rice seedlings where Cd toxicity induced the expression of ABA-regulated genes like pyrroline-5-carboxylate synthase, *proline dehydrogenase*, *betaine-aldehyde dehydrogenase 1* (involved in antioxidant synthesis), *TRAB-1*, *Osem*, *WRKY-71* (encoding transcription factors), and those involved in polyamine biosynthesis [105]. Stimulated antioxidant capacity along with phytostabilization of Cd via adsorption onto root cell wall polysaccharides (due to increased abundance of -OH and -COOH groups) have been reported in plants treated exogenously with ABA [132]. Similarly, ABA supplementation reduced Hg-mediated oxidative injuries in wheat by inducing the expression of cell wall–synthesizing enzymes, increasing cellulose and hemicellulose contents, strengthening root cell walls, and activating antioxidative enzymes [133].

### ABA-antagonistic growth regulators

Other phytohormones like ABA-antagonistic gibberellic acid promoted Cd tolerance in chickpea, rice, and mung bean seedlings by reducing oxidative injuries, activating the ascorbate–glutathione cycle, activating glyoxalases, inducing the expression of cell wall–synthesizing genes, downregulating the expression of Cd transporters, and improving photosynthetic efficiency via crosstalks with NO signaling [134–136]. The ubiquitous pleiotropic growth regulator, melatonin, enhanced Cd tolerance in radish seedlings via differential regulation of 14 micro ribonucleic acids (RNAs), Cd transporters, and activation of the antioxidant machinery [137]. Altered expression of microRNA398ab (regulating *superoxide dismutase*) was observed in melatonin-supplemented plants exposed to Cd [138]. Alongside that, exogenous melatonin activated the defense machinery and improved physiological growth in Cd-stressed wheat, rice, maize, and *Brassica napus*. Nitrosation of melatonin by NO produces NO-melatonin, which might also promote HM tolerance in crops [139].

### NO

The gasotransmitter NO acts downstream of ROS signaling in stressed plants. NO triggers Ca^2+^ fluxes followed by stomatal closure to avoid desiccation, which is the common form of stress faced by the tissues under any suboptimal condition [140]. Exogenous treatment with a NO-releasing chemical, sodium nitroprusside (SNP), or application of the NO synthase inhibitor, N-nitro-L-arginine methyl ester (L-NAME), alleviated U toxicity, reduced H_2_O_2_ production, and improved seedling growth [104]. Application of SNP and L-NAME showed that NO could promote lignin biosynthesis and Hg tolerance in wheat and maize seedlings [106]. Synergistic action of SNP and strigolactone analogue GR24 mitigated Hg toxicity in *Lens culinaris* seedlings by inducing mineral accretion, antioxidants, and glyoxalases, which effectively detoxified ROS and methylglyoxal and reduced electrolyte leakage [141]. Crosstalks between strigolactones and S-nitrosoglutathione reductase–dependent NO/S-nitrosothiol synthesis have been previously reported, which coordinate with karrikin signaling to promote plant growth [142]. Overexpression of rate neuron *NO synthase* in rice seedlings downregulated the expression of *CAL1*, *IRT2*, *NRAMP5*, and *CD1* genes encoding Cd transporters, resulting in reduced Cd uptake and enhanced tolerance in the transgenic lines [143]. Alternatively, exogenous SNP improved Cd adaptation in crops like rice, maize, wheat, *Brassica juncea*, chickpea, and pea by increasing quantum efficiency of photosystem II and recovering cell cycle arrest as well as activating the ascorbate–glutathione cycle, nonenzymatic antioxidant biosynthesis, and crosstalks with stress-responsive pathways dependent on Ca^2+^ [103, 144–147]. The beneficial effects of NO depend on its cellular level, which is strictly monitored by cellular mechanisms because excessive formation of NO might trigger nitro-oxidative stress characterized by protein carbonylation and nitration, as has been observed for wheat roots subjected to In stress [148].

## Consequences of MPs and rare HMs in the food chain

**Fig. 2:**
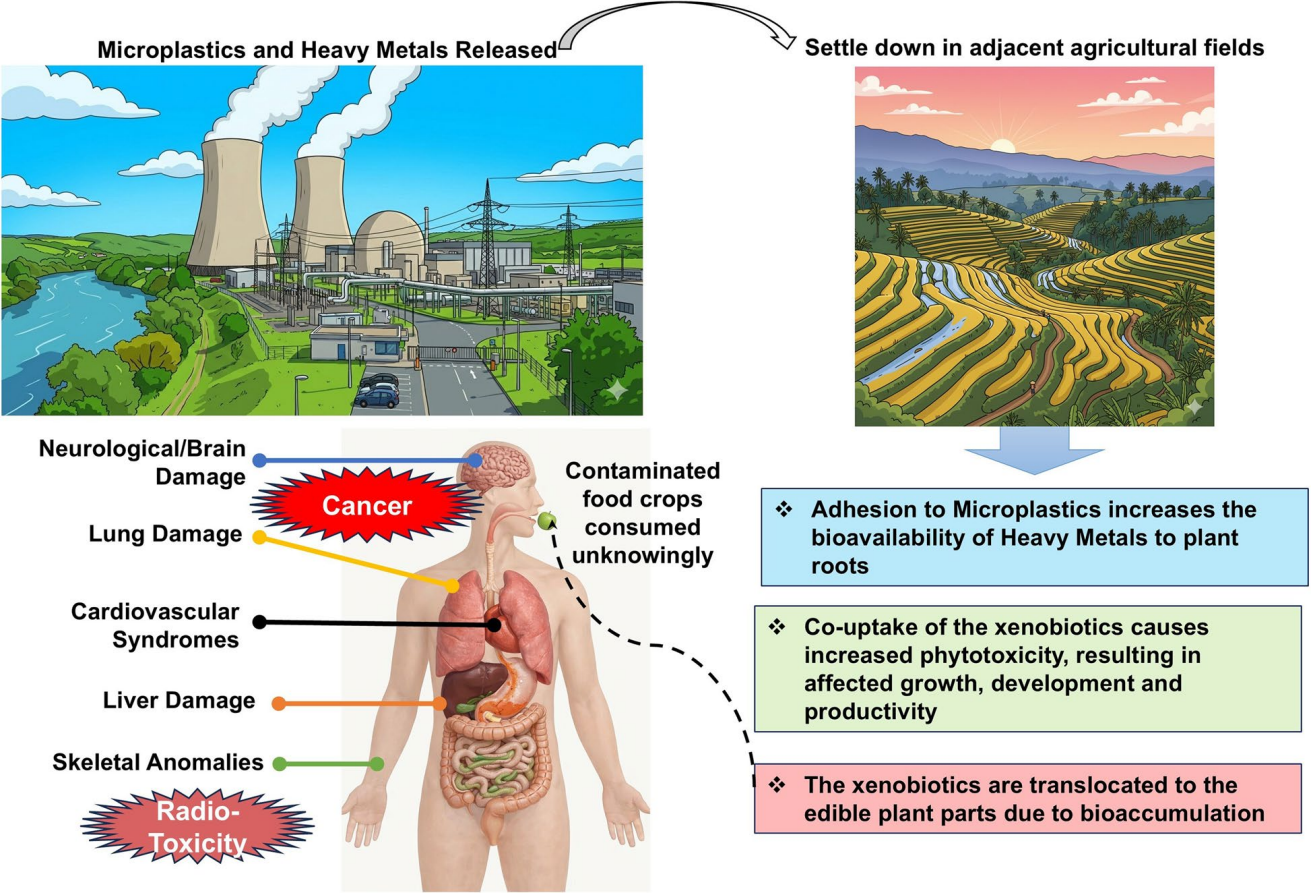
Entry of microplastics and heavy metals into the food chain. Xenobiotics released from nuclear power plants settle over agricultural fields and water sources used for irrigation. Upon bioaccumulation in agricultural crops, these xenobiotics are translocated to edible plant tissues. The local population unknowingly consumes the contaminated plant tissues as staple food or vegetables and are exposed to a large number of complex physiological damages, including cancer

Bioaccumulation of MPs and HMs in edible plant tissues increases the risk of xenobiotic biomagnification and dietary exposure to such severely co-contaminated food, which aggravates physiological anomalies and toxicity in humans and grazing ruminants (Fig. 2). Since this review discusses co-toxicity of MPs and HMs released by NPPs, the primary health concern arises from radioactive poisoning of HMs like U. Ingestion of radioactive U through consumption of polluted food triggers ionizing radiation throughout the human body, targeting cardiac substructures and leading to cardiovascular and cerebrovascular syndromes [149]. Chronic exposure to the α-particles released by radioactive isotopes of U can also promote kidney, liver, and lung damage; bone degeneration; and even cancers [150]. Apart from the radioactive effect, dietary entry of HMs adsorbed to MPs have been known to accelerate the disease progression of a variety of cancers along with irreversible reproductive and developmental anomalies since the xenobiotics bioaccumulate within the adipose tissue [151, 152]. Polystyrene MP and HM co-toxicity in human adenocarcinoma cell lines revealed increased genotoxicity, coupled to DNA breakage and ROS bursts, sufficient to trigger apoptosis. More alarmingly, nontoxic concentrations of polystyrene MPs became highly toxic in presence of HMs due to the mutual adsorption effect, which notoriously enhanced the detrimental effect manifolds [153]. Co-exposure to these xenobiotics reversed cell membrane fluidity, cytoskeletal integrity, and inhibited the ABC transporter activity. Such inhibition was mediated by altered mitochondrial depolarization and compromised ATP synthesis as a result of the coupled effects of polystyrene MPs (not just large-sized particles) and HM [154]. However, we still do not know the bioaccumulative co-capacity of MPs and HMs in the human body, although enhanced accumulation of polyethylene MPs, nickel, and copper was observed in the tissues of earthworms feeding in copolluted soils [155]. This provides a predictive insight into the calamitous consequences of MPs and HMs in agricultural soils and their synergistic, coupled ripple-effects all throughout the food web [156].

## Remote sensing to identify MP and rare HM copolluted sites for avoiding agriculture: the “ephemeral” strategy

Ephemerals are plants with very short life cycles so that they can avoid suboptimal environmental conditions like drought [157]. The ephemeral strategy mentioned in this section refers to an escapist strategy, like these species, following which agricultural sites in and around NPPs that are intensively copolluted with MPs and rare HMs can be identified and avoided for cultivation. HSI-assisted remote sensing can be used as a high-throughput technology to identify the degree of pollution caused by both microplastics as well as HMs in agricultural soils and/or water bodies. Compared with Fourier transform infrared or Raman spectroscopy, HSI reportedly has been used to characterize MPs greater than 250 µm in dimension [158]. In spite of some successes, challenges in HSI circumnavigate around analysis of smaller MPs and development of mathematical models to facilitate streamlined data analysis. Chemometric models currently are being tested to classify pixels acquired through HSI to compress and analyze MP images [159, 160]. Accurate identification of the pixels generated from the particle size and morphology would then ensure poignant recognition of the type of MP polymer [158]. Recently, a multiple regression-dependent inversion model was built up by combining the acquired remote sensing data and successive projections algorithm as a variable selection method to accurately analyze the variable abundance of MPs on surface waters of the Bohai Sea in China [161]. Similar approaches can be adapted to identify MP pollution in riverine water bodies adjacent to NPPs in order to avoid their usage for irrigation purposes. Mathematical modeling involving “convex hull, Gaussian deconvolution, and curve fitting,” and least-squares support vector machine models dependent on THz spectral acquisition can be used to refit the spectral reflectance from MPs and identify them in water bodies (with approximately 80% precision) and soil, respectively [162, 163]. MPs composed of polypropylene, polyethylene terephthalate, methyl methacrylate, and polyethylene have been identified using this strategy in the 800–1000 nm spectral range [162].

Likewise, using the visible near-infrared spectrum of HSI and via vegetation indexing, target HMs can be appreciably detected when present at high levels [164, 165]. It is even more important to remove spectral noise generated from similar spectrally responsive metals and organic materials [166]. Modeling strategies like neural networking and use of partial least-squares and multilinear regression have been combined to sufficiently identify different HMs via remote sensing. Considering the complexity of the environment and the modeling algorithms, the spatial abundance and distribution of HMs in soils have been accurately processed from spectral images acquired by hydrological modeling and analysis platform (HyMAP), Hyperion, and HJ-1A [166]. Cd levels in soil can be detected using infrared (700–900 nm), near-infrared, and far-infrared imaging, whereas Hg can be best identified in the 1140–1200 nm region via HyMAP 51 [167]. In case the proposed agricultural sites are not bare fields, the bioaccumulation of HMs in the existing vegetation also can be estimated to anticipate the extent of pollution in the area. Drawing a correlation between the actual HM concentration and the standardized reflectance spectrum is essential to accurately detect HMs in vegetation. One of the chief waste materials of NPPs, Cd, was detected in the vegetative foliar biomass using spectral imaging optimally at 782 nm [168]. Since both MPs and HMs are detectable by remote sensing technology, this high-throughput method can be used to efficiently locate copolluted sites for avoidance. However, considering the ever-growing population and the question of food security, such an escapist strategy is always not feasible or acceptable to the local population. In that case, remote sensing can be used to identify the copolluted sites, which can then be decontaminated using ecologically sustainable measures of phytoremediation.

## Necessitating bioremediation to pitch-down the agro-plasticosphere

The agro-plasticosphere widely refers to the complex interactions between MPs with abiotic factors like water and soil, biogeochemical cycles, and overall ecological quality, which altogether determine agricultural success. The deleterious impact of the MP cycle on sustainable agriculture has been recently reviewed [169–171]. Enhancement in the plasticosphere around NPPs not only accelerates MP-HM comobilization across soils and water bodies but also increases the translocation of related toxins like bisphenol A along the food web [172, 173]. This directly convulses the agrodiversity, rhizospheric symbiosis, optimal cycling of minerals and nutrients, and, most importantly, crop quality and productivity [174]. Hence, the degradation of environmentally persistent MPs like polyethylene, polypropylene, polystyrene, polyethylene terephthalate, etc. into simplified elements like carbon, sulfur, and nitrogen can be a sustainable way of cleaning and depolluting the agro-plasticosphere. Some of this has been achieved through microbe-assisted bioremediation since some microbes contain and/or release catalytic enzymes capable of breaking down the so-called nonbiodegradable MPs [175].

### Biodegradation of MPs using bacteria and biofilms

Multiple *Bacillus* strains have been found to degrade MPs like polypropylene, polystyrene, and polyethylene terephthalate with efficiencies ranging between 1.6% and 10.7%. Remarkable among them is the ability of *Bacillus* sp. strain YP1, *Bacillus cereus*, and *Bacillus gottheilii* to degrade polypropylene, polyethylene terephthalate, and polyethylene by 10.7%, 7.4%, and 6.2%, respectively, in about 40 days [176, 177]. Bacteria like *Pseudomonas*, *Bacillus*, and *Achromobacter* have been found to degrade polyvinylchloride as well. Strains of *Pseudomonas*, *Bacillus*, *Streptococcus*, and *Staphylococcus*, isolated from soils, have been reported to break down polystyrene and polyethylene terephthalate [178]. The ability of *Pseudomonas aeruginosa* to degrade high-density polyethylene by 20% and polystyrene and polyethylene terephthalate by 10% is striking [179, 180]. The degradation of polyethylene terephthalate was due to the production of carboxylic ester hydrolase by *Pseudomonas aestusnigri* [178].

Most of the bacteria discussed above can effectively survive in soil and rhizosphere; hence, these are great candidates for sustainable degradation of MPs in highly polluted agricultural sites. Some studies further suggest that rather than individual bacteria, the use of biofilms can be more effective to tackle the growing plasticosphere [181]. A MiSeq-based evaluation showed increased abundance of Cyanobacteria, Bacteroidetes, Proteobacteria, and Deinococcota in MP-degrading polymicrobial biofilm communities [182]. Similarly, Bacteroidetes and Proteobacteria were found to be the most prevalent phyla in mixed biofilms that exhibited the potential to degrade MPs [183]. Hydrophobicity of cell surfaces, interaction with soil particles and MPs, and presence of sufficient nutrients is essential for the formation of multispecies bacterial biofilms in soil [184]. It is evident the agricultural sites supporting crop growth and productivity are quite rich in the basic nutrients that are required to support the growth and proliferation of such beneficial biofilms. Therefore, sustainable plans to degrade the agro-plasticosphere requires the optimal use of bacterial bioremediation in situ.

### Biodegradation of MPs using fungi

Fungi can also be used as workable candidates because they can release extracellular multi-enzyme complexes capable of degrading MPs (Table 1). Agricultural soils contain a plethora of fungal species that can be used toward MP bioremediation. In fact, mixed community, interkingdom biofilms formulated with bacteria and fungi do exist, and they are highly bioactive and great candidates of sustainable removal of pollutants from the environment [185]. Polyethylene degradation has been observed with “*Aspergillus niger, Aspergillus versicolor, Penicillium pinophilum, Penicillium frequentan, Penicillium oxalicum*, *Penicillium chrysogenum*, *Acremonium kiliense, Fusarium redolens, Glioclodium virens, Phanerochaete chrysosporium*, and *Verticillium lecanii*” [186, 187]. These fungal species usually degrade and mineralize MPs into usable biomolecules like carbon dioxide, water, and methane, which can be reused by the tricarboxylic acid cycle [188].

## Phytoremediation strategies for rare HM tolerance

HM-hyperaccumulating plant species that are agriculturally unimportant can exist across polluted sites to phytoremediate fields prior to crop cultivation [189]. This strategy has been useful to remove HMs, organic pollutants, and radionuclides since hyperaccumulators have a high bioconcentration factor for the toxicant, due to which the pollutants can be extracted and stored in cellular organelles or detoxified via the internal defense machinery [190]. Phytoremediation can be divided into phytoextraction, phytostabilization, and phytovolatilization [191]. Aquatic plants with large root surface area can also be used for rhizofiltration of HMs from contaminated water sources before using them for irrigation [192]. The extent of bioaccumulation of rare HMs in important crop species have been detailed out in Table 2, and the recent updates on the strategies for phytoremediation that have been successful to remove the rare HMs, released as nuclear discharge, have been represented in Tables 3 and 4.

**Table 2:** Bioaccumulation of rare HMs in economically important crops

HM	Crop species	Bioaccumulation in root (mg kg^−1^)	Bioaccumulation in shoot (mg kg^−1^)	Treatment conditions	References
U	*Brassica juncea*	7,145	⁓380	47.74 mg kg^−1^ U	[193]
	*Helianthus annuus*	136	4.08	82 mg kg^−1^ U	[194]
		⁓315	⁓5.8	318 mg kg^−1^ U	[93]
	*Pisum sativum*	2,327.5	11.16	25 µmol L^−1^ U	[94]
	*Zea mays*	32.01	3.5	50 mg kg^−1^ U	[195]
	*Ipomoea batatas*	2,216	6.67	25 µmol L^−1^ U	[95]
	*Phaseolus vulgaris*	1,243.48	4.15	500 mg L^−1^ U	[196]
Hg	*Oryza sativa* cv. Nipponbare	⁓6,028.03	⁓71.15	25 µmol L^−1^ Hg	[197]
	*O. sativa* cv. Gambiaka Sebela	⁓730	⁓0.89	50 mg kg^−1^ Hg	[198]
	*O. sativa* cv. Sikasso B	⁓820	⁓0.88		
	*Oryza glaberrima* cv. Tog 7102	⁓630	⁓1.3		
	*Brassica napus*	601	286	10 mg L^−1^ Hg	[199]
Co	*Lycopersicon esculentum*	⁓500	⁓200	0.5 mM CoSO_4_	[200]
	*Cicer arietinum*	⁓70	⁓40	400 mg L^−1^ CoCl_2_	[201]
	*Hordeum vulgare* cv. Ea52	⁓4,000	⁓1,900	0.1 mM CoCl_2_	[202]
	*B. juncea*	⁓152,000		0.1 mM CoCl_2_	[203]
Cd	*Triticum aestivum*	⁓600	⁓410	175 mg L^−1^ CdCl_2_	[109]
	*P. sativum*	⁓600	⁓300		
	*L. esculentum*	⁓1200	⁓300		
	*H. annuus*	⁓14	⁓6	Cd-contaminated soil	[204]
	*B. juncea*	⁓23.21 (µmol g^−1^)	⁓13.11 (µmol g^−1^)	200 mg L^−1^ CdSO_4_	[205]
	*O. sativa* (japonica varieties)	⁓0.17		1.2 mg kg^−1^	[206]
	*O. sativa* (indica varieties)	⁓0.3			
In	*O. sativa*	⁓5,000	⁓140	60 mg L^−1^ InCl_3_ for 14 d	[86]
	*T. aestivum* cv. Taichung 2	⁓210	⁓2.8	5 mmol kg^−1^ in soil	[87]
	Cabbage, garlic, water spinach	2–5 (µg kg^−1^)		1.38 mg kg^−1^ In in soil	[207]
Ge	*Lactuca sativa*	⁓1,900		100 mg L^−1^ GeO_2_ (inorganic)	[120]
		⁓2,600		100 mg L^−1^ Ge-132 (organic)	

⁓ denotes “approximately.”

HM: heavy metal

**Table 3:** Recent reports of rare HM depollution via phytoextraction

Species	HM	Specifications	References
*Artemisia serotina*	U	Accumulated 6.22, 2.13, and 8.44 Bq U^238^ kg^−1^ dry weight in roots, stems, and leaves, respectively	[208]
*Lolium perenne*	U, Cd, Hg	Association of the arbuscular mycorrhizal fungus, *Glomus intraradices* increased U extraction in roots of ryegrass; Mycorrhizal consortium and Enterobacteriaceae increased Cd and Hg extraction in ryegrass	[209, 210]
*Cichorium endivia*	Cd	Bioaccumulated 4.5 mg Cd kg^−1^ dry weight when grown in soil containing 42 mg kg^−1^ Cd	[211]
*Theobroma cacao*		Bioaccumulated 3 mg Cd kg^−1^ dry weight when grown in soil containing 2.6 mg kg^−1^ Cd	
*Alchemilla*		Bioaccumulated 8 mg Cd kg^−1^ dry weight when grown in soil containing 16.3 mg kg^−1^ Cd	
*Cynosurus cristatus*		Bioaccumulated 9 mg Cd kg^−1^ dry weight when grown in soil containing 16.3 mg kg^−1^ Cd	
*Hypericum*		Bioaccumulated 3 mg Cd kg^−1^ dry weight when grown in soil containing 16.3 mg kg^−1^ Cd	
*Nicotiana tabacum*		Bioaccumulated 7.48 mg Cd kg^−1^ dry weight by absorbing aerogen Cd via cuticle diffusion, protopast absorption in the stomata when grown in yellow soil	
*Noccaea caerulescens*	Co	Increased Co translocation to leaves upon application of citric acid	[212]
*Astragalus sinicus*		Amendment of soil top-layer with ethylenediamine-N,N’-disuccinic acid increased Co phyto-availability and extraction	[213]
*Alternanthera bettzickiana*		Accumulated 173 mg Co kg^−1^ dry weight when exposed to 440 mg kg^−1^ Co	[214, 215]
*Calotropis procera*		Accumulated 17.5 mg Co kg^−1^ dry weight when exposed to 2.5–14.5 mg kg^−1^ Co	[216]
*Phoenix dactylifera*		Accumulated 18.1 mg Co kg^−1^ dry weight when exposed to 2.0–7.6 mg kg^−1^ Co	
*Miscanthus* × *giganteus*		Accumulated 1.25 mg Co kg^−1^ dry weight roots when exposed to 18 mg kg^−1^ Co	[217]
*Zea mays*	Ge	Inorganic fertilization stimulated Ge extraction by 49%	[218]
*Lupinus albus*		Inorganic fertilization stimulated Ge phytoextraction by 19%	

HM: heavy metal

**Table 4:** Recent reports of rare HM depollution via strategies of phytoremediation other than phytoextraction

Phytostabilization	*Brassica juncea*	U	Slow release of citric acid as root exudates chelated U and increased its uptake; in soils contaminated with 56 mg kg^−1^ U, removal efficiency was ⁓37.35%	[219]
	*Salix* × *aurea pendula* CL’J1011’	Cd	Soil priming with urea increased Cd uptake and stabilized the HM in the plant biomass by increasing the synthesis of nonprotein thiols like glutathione	[220]
	*Arabidopsis thaliana* and tall fescue	Co	Overexpression of gene encoding a DNA binding protein from *Acidithiobacillus ferrooxidans* increased ROS scavenging and tolerance against Co via HM detoxification	[221]
Phytovolatilization	*B. juncea*	Hg	Efficient removal of Hg from water and soil via fixation in roots and stimulated Hg volatilization	[222]
Rhizofiltration	*Lactuca sativa*	U	Accumulation efficiency of 46.9% from polluted water containing 173 µg L^−1^ U	[223]
	*Brassica campestris*		Accumulation efficiency of 77% from polluted water containing 173 µg L^−1^ U	
	*Raphanus sativus*		Enhanced tolerance to high concentrations of U at acidic pH and bioaccumulation of 1,215.8 µg U g^−1^ dry weight of roots	
	*Oenanthe javanica*		Accumulation efficiency of 29.5% from polluted water containing 173 µg L^−1^ U	
	*B. juncea*		20%−23% removal from hydroponic MS medium	[224]
	*Chenopodium amaranticolor*		13% removal from hydroponic MS medium	
	*Eicchornia crassipes*	Cd	⁓60% removal from contaminated site after 15 d of incubation	[192]
	*Spirodela polyrhiza*		⁓50% removal from contaminated site after 15 d of incubation	
	*Pistia stratiotes*		⁓50% removal from contaminated site after 15 d of incubation	
	*Hydrilla verticillate* and *Elodea canadensis*		Keystone rhizobacteria belonging to Pedosphaeraceae and Parasegetibacter promoted Cd uptake	[225]
	*Salvinia natans*	Hg	In Hoagland medium containing up to 0.3 mgHg dm^3^, the bioconcentration factor ranged between 275–780	[226]
	*Eleocharis acicularis*	In	Accumulated 477 mg In kg^−1^ dry weight of roots when grown in contaminated water for 15 d	[227]

⁓ denotes “approximately.”

DNA: deoxyribonucleic acid; HM: heavy metal; MS: Murashige and Skoog media; ROS: reactive oxygen species

## Modern biotechnological approaches to biodegrade MPs and develop rare HM tolerance

### Biotechnology to enhance MP biodegradation

Due to its ubiquitous nature and negative implications on the ecosystem, MP pollution has been gaining worldwide attention, especially after the Covid-19 pandemic, during which unregulated disposal of personal protective equipment kits occurred [228]. Since then, scientific groups have been detecting the injurious health effects of MPs released from multiple industrial sectors, and thus, MP pollution has now become a burning environmental concern. Currently, release of MPs from NPPs results in their deposition into adjacent agricultural fields and water bodies, due to which rare HMs then piggyback on these MPs to accelerate co-toxicity on crops either through direct rhizospheric interactions or after irrigation. Since this problem is rather new, transgenic strategies to develop MP tolerance in plants have not yet been reported [229]. However, modern biotechnological strategies include improved modification of MP-degrading enzymes in microbes for more effective depollution of the contaminated sites. Overexpression of polyethylene terephthalate–degrading enzymes in the autotrophic microalga *Phaeodactylum tricornutum* improved the ability of the transgenic alga to hydrolyze MPs [230]. Heterologous production of manganese-dependent peroxidases in *Escherichia coli* and *Saccharomyces cerevisiae* BY4741 and laccase enzymes in *P. chrysosporium* and *E. coli* enhanced their ability to hydrolyze polyethylene terephthalate [231, 232]. Further studies involving polyethylene terephthalate degradation include development of an improved version of cutinase capable of hydrolyzing the MP within a reduced timeframe of 6.2 h compared with 41.8 h by the nonmodified version of the enzyme [233]. Knowledge of biotechnology has also been applied to increase MP capture and adsorption to microbial surfaces for effective bioremediation rather than biodegradation. Deletion of the *wspF* gene in *P. aeruginosa* stimulated the synthesis of exopolymeric complexes, which, due to their inherent sticky nature, could bind to surrounding polyvinyl chloride MPs like an adhesive. For effective bioremediation, release of these bio-adsorbed MPs is also necessary after collecting the bacterial biofilm consortia from the polluted sites. To solve this issue, the same bacteria was engineered to transcribe the *yhjH* gene under an arabinose-inducible promoter and so only under arabinose-mediated induction the bacteria expressed *yhjH*, which reduced the endogenous content of cyclic dimeric guanosine monophosphates and release of the bound MPs [234]. This biotechnological “capture and release” system would be ideal for bioremediation of MPs and reuse of the biofilm population to scavenge more MPs.

### Bioinformatics as a resource tool to develop MP bioremediation

Bioinformatics has long supported metabolic engineering studies to marginalize the gap between the genetic diversity of MP-degrading microbial enzymes and their predicted efficacy for biodegradation [235]. Although extensive wet lab investigations are still required to testify the predicted claims in bioinformatic databases, such in silico tools are very helpful in designing combinatorial approaches involving genetic-metabolic engineering and systems biology for effective bioremediation of MPs. Supportive in silico databases like the University of Minnesota Biocatalysis/Biodegradation Database, the Environmental Contaminant Biotransformation Pathway Resource (enviPath), MetaCyc database, and BioCyc database provide precise information on the genes and processes associated with the metabolic pathways that promote biodegradation of MPs [229]. Machine learning and computational modeling can anticipate metabolic circuits capable of hydrolyzing and detoxifying MPs [236].

### Transgenic strategies to develop rare HM tolerant plants

Unlike with MPs, multiple attempts have been taken to incorporate and overexpress transgenes in crops so as to enable their unhindered growth in areas polluted with HMs. Tables 5 and 6 summarize the most recent advances in generating crop tolerance against Cd and the other rare HMs, respectively, spewed out by NPPs. So with the current technology at hand, the most effective strategy to counter MP–rare HM copollution would be use of natural or engineered microbes to degrade MPs and then grow tolerant crop genotypes that are unaffected by the residual HM toxicity. Overexpression of transgenes encoding transporters or osmotic stress-responsive transcription factors or other related signaling molecules effectively reduce the soil-to-biomass translocation of specific HMs and also refine the overall defense machinery, comprising osmolytes and nonenzymatic and enzymatic antioxidants, due to which ROS are effectively scavenged and oxidative and physiological injuries are mitigated. However, due to potential occurrence of gene escape, cultivation of transgenic plants faces several regulatory barriers. Unwanted gene flow from transgenic plants can disrupt ecological balance by creating superweeds or harming nontarget organisms. Thus, additional care must be taken in accordance with the governmental guidelines to use transgenics for HM tolerance.

**Table 5:** An update on the transgenic approaches to develop crop tolerance against cadmium

Rare HM	Target species	Transgene	Effects	Reference
Cd	*Glycine max*	Transcription factor encoded by *WRKY172* from soybean itself	Overexpression reduced the translocation of Cd to the aerial biomass. The transgenic plants encountered lowered oxidative injuries and malondialdehyde production due to high production of flavonoids, lignins, and increased activity of peroxidases	[237]
	*Solanum lycopersicum*	*RING1*, encoding a RING E3 ligase, from tomato	Overexpression boosted Cd tolerance due to activation of the antioxidative ascorbate–glutathione pathway and *phytochelatin synthase* expression. Overall photosynthetic performance was well-maintained in the stressed transgenic plants	[238]
	*Malus domestica*	*IAA24*, involved in auxin signaling, from apple seedlings	Overexpression reduced ROS formation and promoted Cd tolerance in transgenic apple seedlings due to increased activity of enzymatic antioxidants and steady maintenance of the photosynthetic activity	[239]
	*Populus trichocarpa*	*AREB3*, encoding a transcription factor involved in ABA signaling, from poplar seedlings	Overexpression efficiently detoxified Cd and generated tolerance; it also increased the dry biomass and photosynthetic efficiency in transgenic poplar lines	[240]
	*Solanum tuberosum*	Transcription factor encoded by *WRKY2* from *Vitis vinifera*	Overexpression led to efficient sequestration of Cd within plant roots. Overall malondialdehyde formation was reduced due to increased activity of superoxide dismutase and catalase enzymes	[241]
	*Populus euphratica*	Cd resistance protein encoded by *PCR10* from poplar	Overexpression stimulated seedling growth and development and promoted tolerance against both Cd and Al stresses. Leakage of osmolytes and ions was reduced and photosynthetic efficiency was enhanced in these stressed transgenics	[242]
Hg, Cd	Yeast	Vacuolar transporter encoding genes like *ABCC1* and *ABCC2* from *Arabidopsis*	Overexpression increased channelization of Hg and Cd to vacuoles, resulting in enhanced tolerance toward the HM	[76]

ABA: abscisic acid; HM: heavy metal; ROS: reactive oxygen species

**Table 6:** An update on the transgenic approaches to develop crop tolerance against other rare HMs released by NPPs

Rare HM	Target species	Transgene	Effects	Reference
U	*Nicotiana tabacum*	*Uranyl reductase* from *Desulfovibrio vulgaris*	Heterologous overexpression in tobacco plants ensured normal phenotypic growth even in presence of uranyl ions in growth medium	[243]
Hg	*Arabidopsis thaliana*	Mercuric transporter encoding gene *merT* from *Pseudomonas alcaligenes*	Overexpression increased Hg tolerance due to increased activity of enzymatic antioxidants like superoxide dismutase, catalase, and guaiacol peroxidase, which effectively detoxified ROS and reversed oxidative injuries like thiobarbituric acid production	[244]
	*A. thaliana* and *N. tabacum*	*merA* and *merB*	The transgenic plants overexpressing the genes exhibited unhindered growth and productivity even when grown under high Hg stress. The edible plant parts also bioaccumulated less Hg and were found to be safe for consumption	[245]
	*Solanum lycopersicum*	Genes belonging to the ascorbate–glutathione cycle were stacked in a single cassette under the influence of the stress-inducible *Rd29A* promoter	The overexpressing lines exhibited increased photosynthetic efficiency, gas exchanges, and antioxidant enzyme activity, along with reduced anatomical damages (as revealed through electron microscopy) and electrolyte leakage during Hg stress, resulting in enhanced tolerance	[246]
	*Oryza sativa*	Gene (*MTH1745*) from *Methanothermobacter thermoautotrophicum* encoding a protein disulfide isomerase-like protein exhibiting chaperone function and disulfide isomerase activity	Overexpression reduced the production of ROS and malondialdehyde along with concomitant increase in superoxide dismutase and peroxidase activity. Level of nonprotein thiols, including reduced glutathione, also increased, which explained effective chelation and detoxification of Hg	[247]
	Poplar	A tau class of glutathione-S-transferase encoding gene GSTU51 from the hybrid poplar clone (*Populus alba x Populus tremula* var*. glandulosa* Clone BH1)	Overexpression reprogrammed the defense machinery and stimulated selective tolerance against Hg and methyl viologen stress	[248]
	*A. thaliana*	Gene encoding caffeic acid *O*-methyltransferase (COMT) from *Lycoris aurea*, which is known to catalyze lignin biosynthesis	Overexpression of the transgene elevated the level of antioxidants and stimulated the activity of antioxidative enzymes, which scavenged ROS and promoted Hg tolerance	[249]
	*A. thaliana* and *Populus trichocarpa*	ATP-binding cassette transporter gene, *ABCC1* from *P trichocarpa*	Overexpression increased the uptake of Hg by ⁓72% in *Arabidopsis* and by ⁓160% in poplar. This strategy could be used to generate noncrop plants capable of mediating Hg phytoextraction and decontamination	[250]
Co	Yeast	*Cd-induced protein AS8* (*CIPAS8*) from *Populus euphratica* and *Salix linearistipularis*	Overexpression reduced the uptake of Co and promoted tolerance through maintenance of intracellular Co homeostasis	[251]

⁓ denotes “approximately.”

HM: heavy metal; NPP: nuclear power plant; ROS: reactive oxygen species

## Conclusion and future perspectives

**Fig. 3:**
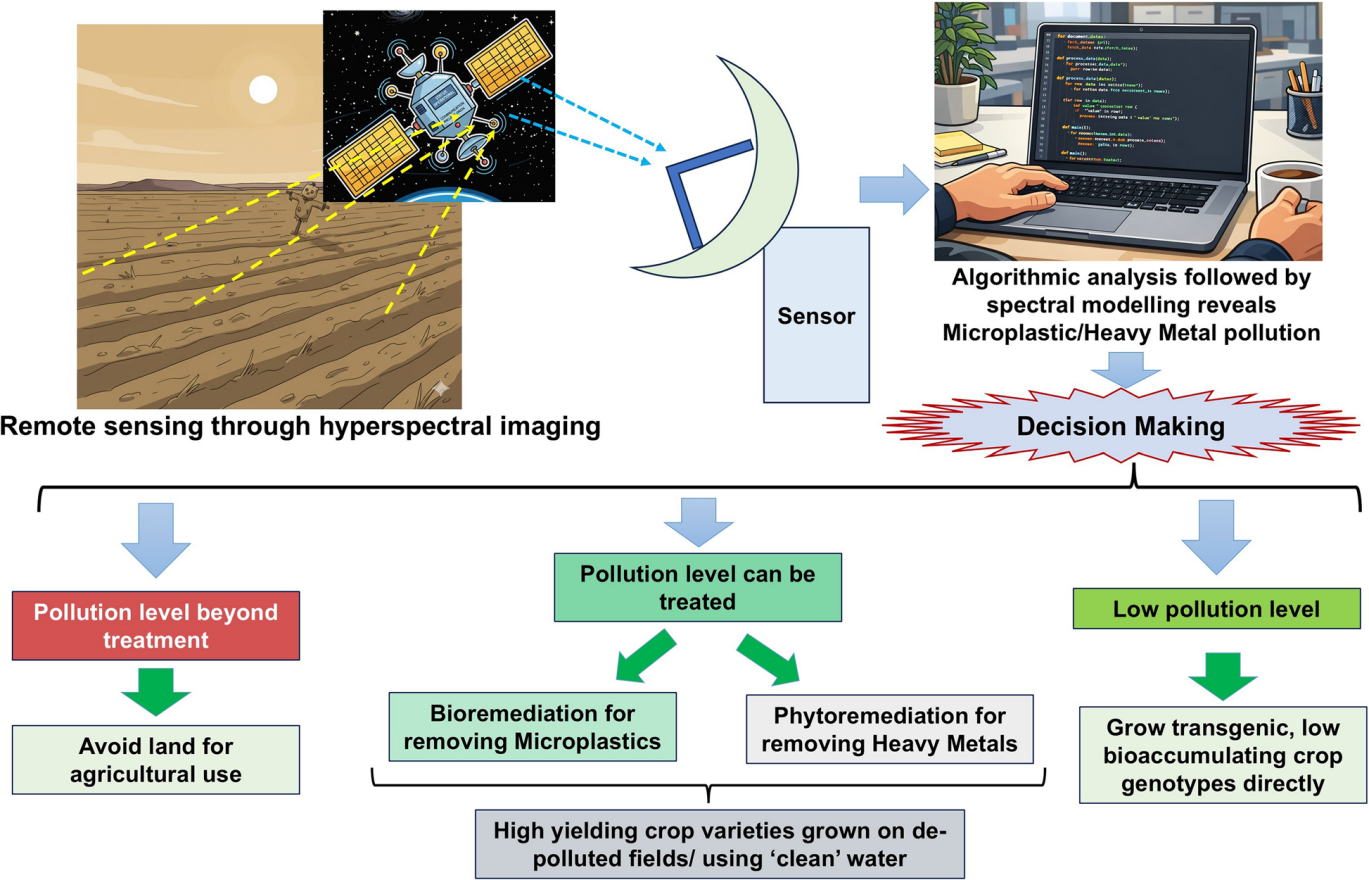
Hyperspectral imaging-based remote sensing can detect microplastic/heavy metal pollution in agricultural fields or water bodies. This knowledge can be provided to a decision-making body that, based on the extent of pollution, could avoid agriculture activity in that area or adopt sustainable strategies to depollute the site. If the level of pollution is low, then genetically engineered, low xenobiotic bioaccumulating genotypes could be cultivated directly. This decision-making policy would be useful to stop the entry of xenobiotics into the food chain to ensure pollutant-free food crops for the local population

Compromised growth physiology and marginalized productivity in crops and vegetables cultivated in areas polluted with MPs and rare HMs like U, Cd, Hg, Co, Ge, and In, which are typically released as nuclear wastes, must be considered as a significant ecological concern. Although several studies have exemplified the toxic effects of individual HMs or MPs on various crops and vegetables, no study has been performed to understand the combined phytotoxic effects of these multiple HMs present in nuclear wastes. This text primarily upholds how the rare HMs can piggyback on MPs via adsorption and act in combination as an emerging class of pollutant in agricultural fields adjacent to NPPs. These pollutants bioaccumulate in plants and crops through various active and passive routes and then exert severe phytotoxicity, which is mainly manifested as oxidative damages. In response, plants activate multiple phytohormonal signaling pathways to tackle such toxicity, but the susceptible genotypes usually succumb in the long run. Thus, multiple strategies using remote sensing, bioremediation, and genetic engineering can be used to avoid or depollute the contaminated sites or grow tolerant genotypes directly at the pollutes sites (Fig. 3). The transporters associated with uptake of the pollutants can be biotechnologically downregulated or particular phytohormonal signaling pathways can be strengthened via gene overexpression to generate transgenic lines capable of tolerating multi-HM and MP stress. Furthermore, species that have been tested for bioremediating MPs and HMs should be screened in contaminated areas surrounding NPPs so that a few hyperaccumulators capable of significantly removing more than one type of HM along with MPs can be identified. This text also emphasizes the need for intricate pangenomic studies involving genomics, transcriptomics, proteomics, and metabolomics in crops and vegetables grown in the vicinity of NPPs so that the synergistic toxic effect of MPs and multiple rare HMs can be monitored at the molecular level.

**Fig. 4:**
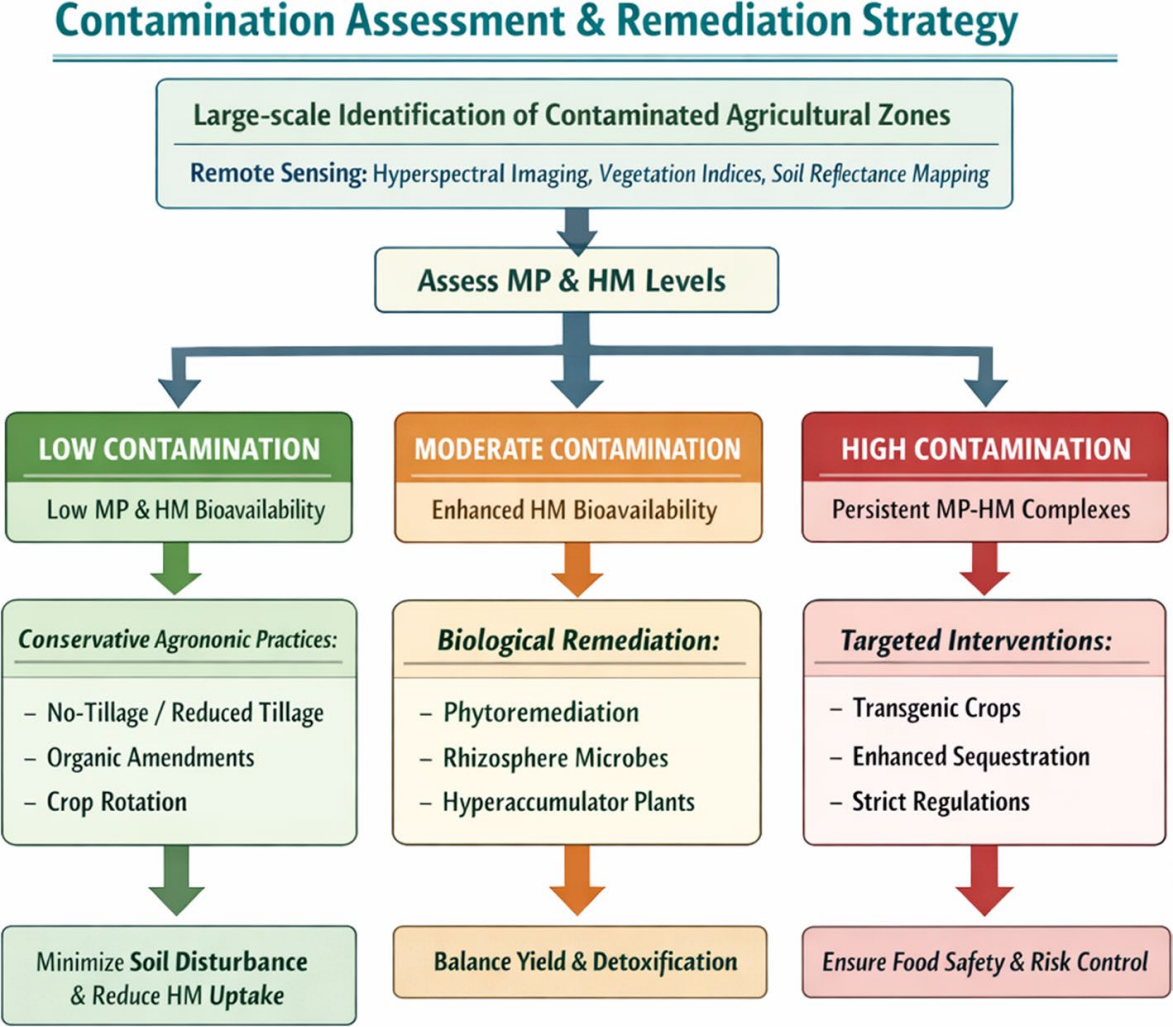
The decision flowchart for contamination assessment and remediation strategy. The following three approaches can be adopted under three different situations: 1) When contamination level is low, as characterized by less HM bioavailability and limited MP-HM complex formation, conservative agronomic interventions may be used. These include no-tillage or reduced-tillage practices, organic amendments, and crop rotation, which collectively minimize soil disturbance, limit further MP mobilization, and reduce HM uptake by crops. 2) When contamination level is moderate and MPs significantly enhance HM bioavailability but crop productivity remains viable, biological remediation strategies can be warranted. Phytoremediation using tolerant or hyperaccumulator plant species, supported by rhizospheric microbes or engineered consortia, can reduce soil metal loads while maintaining agroecosystem function. At this stage, remediation efforts aim to balance yield preservation with gradual detoxification. 3) When contamination level is high, particularly in areas proximal to NPPs where persistent MP-HM complexes pose long-term risks to food safety, targeted interventions are recommended. These include the controlled deployment of transgenic crops with reduced HM uptake, enhanced sequestration capacity, and improved stress tolerance. Such approaches should be accompanied by strict regulatory oversight and site-specific risk assessment. HM: heavy metal; MP: microplastic

To translate the mechanistic insights discussed in this review into actionable strategies, a decision-oriented framework that integrates contamination assessment with remediation goals is proposed. The first step involves large-scale identification of contaminated agricultural zones using remote sensing approaches—including HSI, satellite-derived vegetation indices, and soil reflectance mapping—to estimate both MP burden and associated HM accumulation. The following approaches can be adopted under three different situations (Fig. 4):

1. When contamination level is low, as characterized by less HM bioavailability and limited MP-HM complex formation, conservative agronomic interventions may be used. These include no-tillage or reduced-tillage practices, organic amendments, and crop rotation, which collectively minimize soil disturbance, limit further MP mobilization, and reduce HM uptake by crops.
2. When contamination level is moderate and MPs significantly enhance HM bioavailability but crop productivity remains viable, biological remediation strategies can be warranted. Phytoremediation using tolerant or hyperaccumulator plant species, supported by rhizospheric microbes or engineered consortia, can reduce soil metal loads while maintaining agroecosystem function. At this stage, remediation efforts aim to balance yield preservation with gradual detoxification.
3. When contamination level is high, particularly in areas proximal to NPPs where persistent MP-HM complexes pose long-term risks to food safety, targeted interventions are recommended. These include the controlled deployment of transgenic crops with reduced HM uptake, enhanced sequestration capacity, and improved stress tolerance. Such approaches should be accompanied by strict regulatory oversight and site-specific risk assessment.

Collectively, this tiered decision framework provides a rational basis for selecting remediation strategies according to pollution severity and remediation objectives. By aligning molecular mechanisms with field-scale management decisions, the framework underscores how MP-guided HM toxicity research can inform sustainable agricultural practices in radiation-adjacent environments.

## Data Availability

All data presented in the manuscript are available from the cited references.
